# Spectral Algal Fingerprinting and Long Sequencing in Synthetic Algal–Microbial Communities

**DOI:** 10.3390/cells13181552

**Published:** 2024-09-14

**Authors:** Ayagoz Meirkhanova, Sabina Marks, Nicole Feja, Ivan A. Vorobjev, Natasha S. Barteneva

**Affiliations:** 1 School of Science and Humanities, Nazarbayev University, Astana 010000, Kazakhstan; ayagoz.meirkhanova@nu.edu.kz (A.M.); ivan.vorobyev@nu.edu.kz (I.A.V.); 2 Faculty of Biology, University of Duisburg-Essen, Campus Essen, 45141 Essen, Germany; sabina.marks@uni-due.de (S.M.); nicole.feja@uni-due.de (N.F.); 3 National Laboratory Astana, Nazarbayev University, Astana 010000, Kazakhstan; 4 The Environmental Research and Efficiency Cluster, Nazarbayev University, Astana 010000, Kazakhstan

**Keywords:** synthetic algal–microbial communities, spectral flow cytometry, imaging flow cytometry, long sequencing, nanopore-based sequencing

## Abstract

Synthetic biology has advanced in creating artificial microbial and algal communities, but technical and evolutionary complexities still pose significant challenges. Traditional methods, like microscopy and pigment analysis, are limited in throughput and resolution. In contrast, advancements in full-spectrum cytometry enabled high-throughput, multidimensional analysis of single cells based on size, complexity, and spectral fingerprints, offering more precision and flexibility than conventional flow cytometry. This study uses full-spectrum cytometry to analyze synthetic algal–microbial communities, enabling rapid species identification and enumeration. The workflow involves recording individual spectral signatures from monocultures, using autofluorescence to capture populations of interest, and creating a spectral library for further analysis. This spectral library was used for the analysis of the synthetic phytoplankton communities, revealing differences in spectral signatures. Moreover, the synthetic consortium experiment monitored algal growth, comparing results from different instruments, highlighting the advantages of the spectral virtual filter system for precise population separation and abundance tracking. By capturing the entire emission spectrum of each cell, this method enhances understanding of algal–microbial community dynamics and responses to environmental stressors. The development of standardized spectral libraries would improve the characterization of algal communities, further advancing synthetic biology and phytoplankton ecology research.

## 1. Introduction

In recent decades, synthetic biology has made significant progress in building artificial microbial and algal communities [1,2]; however, the difficulty of engineering complex biological systems remains significant due to technical hurdles and the intricate nature of biosystems, which evolve and refine themselves over time [3]. Community selection experiments address fundamental questions in ecology and evolution, as well as applied biotechnological issues, yet constructing more complex stable communities to study the effects of different bacteria on community dynamics remains challenging. Some of the challenges include the fact that unialgal systems are not stable, and bacterial contamination is likely if the algal community is maintained heterotrophically on a medium with a fixed source of carbon [4] or in the presence of dead cells. Several studies have demonstrated that algal–microbial synthetic consortia, which exhibit mutualism, can be engineered [5,6,7]. However, algal–microbial consortia development is usually limited by studying a few algae (often, systems are over-dominated by certain algae) or only the bacterial part of the consortia, with some exceptions [8,9]. Moreover, the long-term homeostasis of algal–microbial consortia could be challenging to maintain since evolutionary constraints are known to limit the long-term stability of synthetic systems [10], and behavior and the composition of engineered communities are “unpredictable” [11].

Finally, the lack of methods to track the dynamics of mixed algal–microbial systems further complicates the development of complex synthetic systems. Identification and characterization of individual microalga and bacteria in the consortium are essential for engineering such consortia and the development of subsequent applications [12]. While microbial community composition analysis has advanced significantly, including the classification of reads up to the species level [13], effective methods for plankton identification and characterization are still limited. Traditionally, microbiome analysis in such systems was conducted at the genus and class levels due to limitations in next-generation sequencing. However, with the development of long sequencing techniques, more detailed analyses have become possible. For instance, in a long-term mesocosm experiment, species-level visualization using imaging cytometry combined with long sequencing was employed [14]. Integrating a full-spectrum approach would provide a distinct advantage by enabling more precise and comprehensive analysis.

An algal community can be categorized into subgroups based on morphology, size, cellular functions, and interactive relationships. However, morphology-based taxonomy has limitations, particularly in distinguishing cell types with similar morphologies. Traditional methods for studying microbial and algal communities, such as microscopy and pigment analysis, are constrained by throughput, taxonomic resolution, and functional characterization, primarily differentiating specific taxonomic groups. Spectrofluorimetry, spectroscopy, and HPLC-based approaches provide averaged integrated data at a population level [15,16,17,18,19]. The maximum of taxonomic groups identified by HPLC pigment analysis is typically limited to 4–7 distinct groups [20,21]. While full-resolution reflectance spectra are used in discriminant and classification analyses of algae by airborne hyperspectral sensors, applying spectral unmixing algorithms based on reference data [21,22,23,24], there is still a need for an adequate in situ method. Conventional flow cytometry struggles to distinguish cells from other particulate matter in the water matrix, and imaging flow cytometry can differentiate only a few fluorescent channels. In contrast, full-spectrum cytometry has recently transformed the field by offering high-throughput, multidimensional analysis of single cells based on their size, complexity, and spectral fingerprint [25,26,27].

This study introduces full-spectrum cytometry as a powerful tool for analyzing synthetic algal–microbial communities, facilitating rapid, high-throughput analysis from species identification to enumeration without extensive sample preparation. By capturing the complete emission spectrum of each cell, full-spectrum cytometry provides valuable insights into phytoplankton community composition, structure, and dynamics, as well as their responses to environmental stressors that influence community structure [28,29,30]. Algal cells, distinguished by autofluorescent spectral signatures, are separated taxonomically, while light scattering parameters assess cell size and complexity, distinguishing target events from debris. Spectral cytometry identifies highly autofluorescent subpopulations using virtual filtering [28] or only an autofluorescence finder algorithm [31]. Characterizing microbiota associated with algae from in-house cultures or natural consortia is crucial as microalgae growth phases correlate with shifts in microbial phylotypes [32], although microbial dynamics in synthetic systems remain understudied. This research demonstrates that modern spectral cytometry, coupled with species-level 16S rRNA sequencing, effectively identifies and enumerates algae based on their spectral signatures, advancing our understanding of phytoplankton ecology, synthetic biology, and evolution.

## 2. Materials and Methods

### 2.1. Sample Preparation and Instrument Setup

Algal cultures (Table 1) were obtained from the Culture Collection of Algae at the University of Göttingen (Germany) and the Central Collection of Algal Cultures at the University of Duisburg-Essen (Germany). Cultures were maintained in Basal Medium (BM) at 21 °C, under a 12/12 L/D cycle in algae growth chamber AL-30L2 (Percival, Perry, IA, USA). Samples were recorded in parallel on three instruments: 7-laser ID7000 spectral cell analyzer (Sony Biotechnology, San Jose, CA, USA), 5-laser BD FACSAria Special Order cell sorter (BD Biosciences, Franklin Lakes, NJ, USA), and 4-laser ImageStreamX MarkII (Amnis–Cytek, Fremont, CA, USA) imaging flow cytometer equipped with 40× and 60× objectives [33]. Samples prepared for cytometric analysis included mono- and mixed cultures and were recorded using a standard 24-tube rack. Each sample was placed in round-bottom 5 mL tubes, with a minimum volume of 250 µL, and arranged in specified positions on the rack. Prior to data acquisition, instrument settings were adjusted and set in accordance with each sample (FSC and SSC gains depending on algal culture, threshold value—11%, PMT voltage ranging from 40% to 70%). Samples were recorded at a minimum flow rate (=1), and 50,000 events were set for data acquisition. FSC_A vs. SSC_A plots were used for the initial assessment of instrument settings in the “Preview” mode prior to recording.

Next, the synthetic consortium was set up and included representatives of three major phytoplankton groups—Cyanobacteria (*Microcystis* sp., *Synechococcus* sp., *Synechocystis* sp.), Chlorophyta (*Chlamydomonas* sp., *Scherffelia* sp., *Haematococcus pluvialis*), and Cryptophyta (*Rhodomonas* sp., *Cryptomonas* sp., *Chroomonas* sp.)—with size ranging from 2 to 25 µm (Table 1, Figure S1), growth of which was monitored daily for the period of 9 days. Triplicates were also analyzed in parallel using BD FACSAria SORP cell sorter (BD Biosciences, Franklin Lakes, NJ, USA) and ImageStreamX MarkII (Amnis–Cytek, Fremont, CA, USA) imaging flow cytometer. The microbial community composition during the last day of the experiment was assessed using nanopore-based full-length 16S rRNA gene sequencing.

### 2.2. Autofluorescence Finder Tool and Virtual Filters Setup

The ID7000 software’s vs.2.0.2 Autofluorescence Finder Tool was used to analyze the algae samples, treating autofluorescence as an independent fluorescent parameter. This feature is crucial for the taxonomic identification of different algae. By incorporating excitation lasers ranging from 320 nm to 808 nm, we included unique emission regions in the algal spectral signature (fluorescence intensity and shape), distinct from the chlorophyll peak and highly variable among different algae. This setup allowed for the detection of individual autofluorescent spectral signatures for each of the recorded algal monocultures. Following the identification of the best separation between autofluorescent populations, subpopulations of interest were gated and examined for their spectral signatures. Each population demonstrated a unique autofluorescence spectrum. Furthermore, the “Autofluorescence Finder” tool was used to set up optimized virtual filters. In a similar manner, highly variable regions between different algal taxonomic groups were defined and used to set a pair of virtual filters (VFs). Defined VFs are next used to separate subpopulations of interest within multi-component mixes.

### 2.3. Data Analysis

Initial cytometric data analysis and population identification were performed using the ID7000 software. Identification of subpopulations within an artificial mix involved consecutive gating steps using a combination of 2D plots with specified virtual filters for best separation. In addition, t-distributed Stochastic Neighbor Embedding, otherwise known as t-SNE, was performed using FCS Express vs.7.18 (De Novo Software, Pasadena, CA, USA). A modified version of t-SNE–opt-SNE, a tool allowing the mapping of high-dimensional cytometry data onto a two-dimensional plot while conserving the original high-dimensional structure, was used to independently identify subpopulations within the artificial mix, minimizing gating bias. Obtained clusters were then used to compare data acquired on different cytometers. To visualize fluorescence intensity dispersion, the coefficient of variation was calculated and plotted across all detection channels.

### 2.4. Sequencing Library Preparation and Bioinformatic Analysis

Library preparation, the sequencing run on the MinION Mk1C device, and bioinformatic analysis were performed in the laboratory following the end of a 9-day synthetic consortium experiment. Firstly, DNA was extracted using the DNEasy Power Water Kit (Qiagen, Hilden, Germany) according to the manufacturer’s instructions, with an additional heating lysis step. 16S rRNA gene sequencing library was prepared using SQK-16S024 kit (https://nanoporetech.com/document/chemistry-technical-document, accessed on 26 August 2024), Oxford Nanotechnologies (ONT) (UK), Oxford, UK, which utilizes universal 16S primer pair 27F (5′-AGAGTTTGATCCTGGCTCAG-3′) and 1492R (5′-TACGGYTACCTTGTTACGACTT-3′) to amplify full-length 16S rRNA gene [34,35]. The PCR reaction mixture included nuclease-free water, input DNA, LongAmp Hot Start Taq 2X Master Mix (New England Biolabs, Ipswich, MA, USA), and the respective 16S barcode primer. PCR products were next purified using AMPure XP beads (Beckman Coulter, Brea, CA, USA). In the final library preparation step, sequencing adapters were added to the barcoded samples. Raw data underwent basecalling using the guppy basecaller (ONT, UK). The resulting FASTQ files were processed using Python algorithms, beginning with read assessment, followed by quality (Q-score > 10) and read length filtering. The next steps involved adapter sequence removal and demultiplexing of reads into their respective barcodes. The demultiplexed reads were then classified using the Emu taxonomic abundance estimator [13] along with a custom reference database [36] up to the species level (https://gitlab.com/treangenlab/emu, accessed on 10 April 2024). Lastly, the data were rarefied before further analysis.

## 3. Results

### 3.1. Construction of a Spectral Signature Library for Phytoplankton Groups

**Figure 1:**
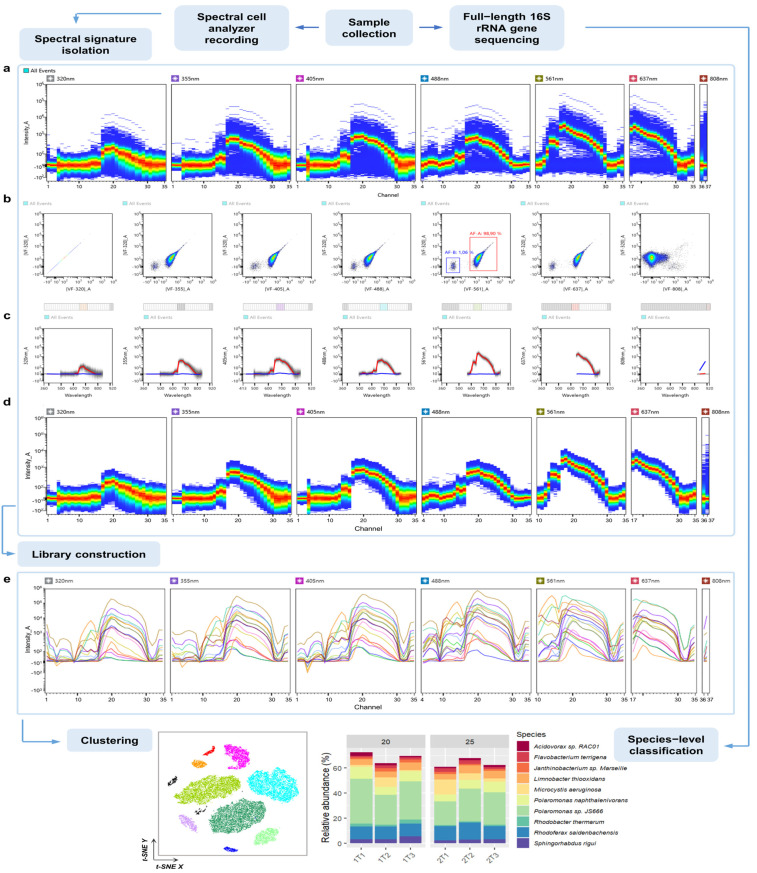
Schematic overview of the proposed experimental workflow for the analysis combining full-spectrum cytometry and sequencing. The workflow involves parallel analysis of obtained samples using a MinION Mk1C-based sequencing platform (ONT) and ID7000 spectral cell analyzer (Sony Biotechnology). The first step in the analysis of spectral data requires (**a**) recording and isolation of single spectral signatures, for which the“Autofluorescence Finder” tool is utilized. During this step (**b**), a set of optimal virtual filters for discrimination autofluorescent populations is determined. As defined using the tool, AF-A corresponds to *Microcystis* sp. and AF-B to debris within the sample; (**c**) respective emission spectra of two defined autofluorescent populations displayed below (red line—AF-A, blue line—AF-B). (**d**) Single spectral signature is then extracted, and (**e**) a library containing unique spectral signatures corresponding to respective phytoplankton species is then constructed and utilized during subsequent analysis, including unsupervised clustering techniques (e.g., t-SNE).

The first step in the analysis of complex communities requires the construction of a library with unique spectral signatures for different phytoplankton groups. For this purpose, single phytoplankton cultures were recorded first on an ID7000 spectral cell analyzer (Sony Biotechnology), and individual spectral signatures were then extracted. Figure 1 demonstrates the general workflow for the analysis of *Microcystis* sp. monoculture as an example. During the recording, emission spectra across seven lasers were obtained with the presence of background noise/cellular debris (Figure 1a). Autofluorescence was then utilized in order to discriminate the spectra of interest from the debris/noise. In some cases, direct gating based on light scattering is possible as well; however, for picoplankton (e.g., *Synechococcus*), with cell size ranging from 0.2 to 2 µm, the gating is not precise. Instead, intrinsic cell fluorescence was used to distinguish events of interest from low-intensity/no-intensity events. With the“Autofluorescence Finder” tool, clear separation between the two autofluorescent populations was observed across all lasers, except for 808 nm, given the optimal set of virtual filters (VFs). Figure 1b,c demonstrate pairwise density plots of [VF-320]_A against VFs of six remaining excitation lasers as an example, with the detection of two populations: AF-A—*Microcystis* sp.—98.9% and AF-B—debris—1.06% ([VF-320]_A vs. [VF-561]_A). In this case, filters were set around channel 20, where emission intensity was the highest. The unique spectral signature for *Microcystis* sp. was then extracted by plotting the emission of autofluorescent population AF-A on a ribbon plot (Figure 1d). The same workflow was applied for the acquisition of spectral signatures of the remaining algal monocultures (Figure 1e), resulting in a spectral library used for further analysis.

### 3.2. Autofluorescence-Based Discrimination of Multi-Component Suspensions

**Figure 2:**
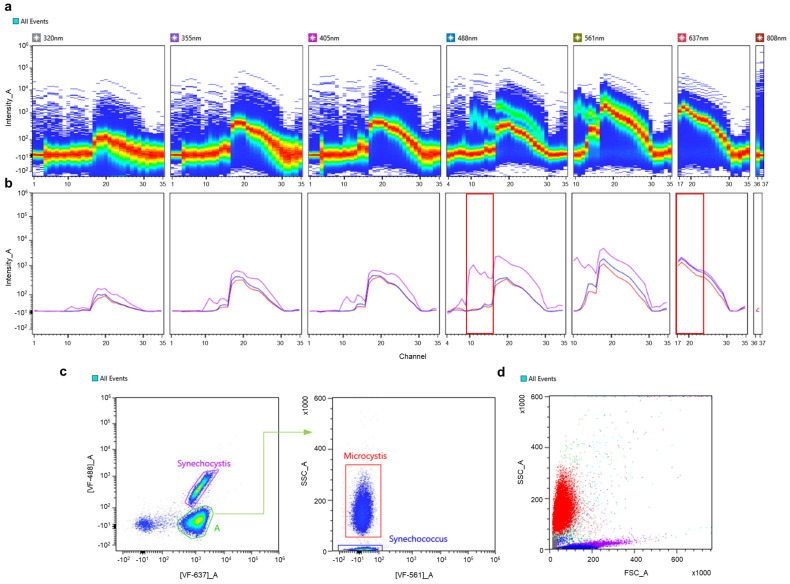
Discrimination of cyanobacteria in a synthetic mix based on autofluorescence. (**a**) Ribbon plot demonstrating combined spectral signatures of three cyanobacterial representatives across 7 excitation lasers: *Synechococcus* sp., *Synechocystis* sp., and *Microcystis* sp. (**b**) Overlay plot of individual spectral signatures for each cyanobacterial representative; red regions indicate regions of interest, used for setting an optimal pair of VFs. (**c**) Dot plot of **[VF-637]_A** against **[VF-488]_A** with two autofluorescent populations identified: *Synechocystis* sp., population A was then plotted with **[VF-561]_A** against **SSC_A** to resolve two subpopulations of *Microcystis* sp. and *Synechococcus* sp. (**d**) Scatter plot (**FSC_A** against **SSC_A**) demonstrates relative position of each identified cyanobacterial species. Color coding: (*Synechococcus* sp.—blue, *Synechocystis* sp.—violet, and *Microcystis* sp.—red).

**Figure 3:**
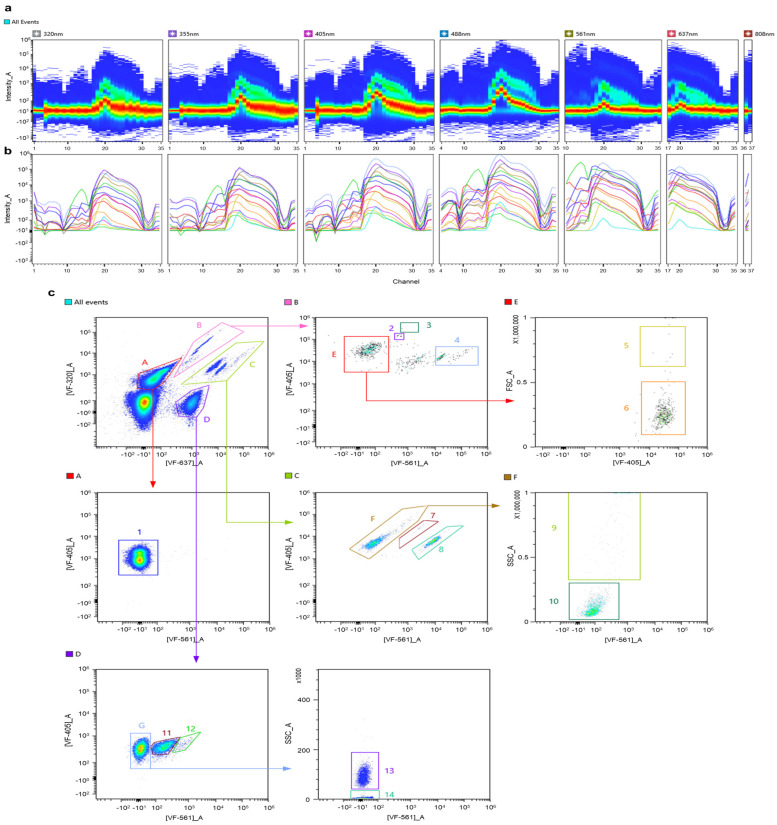
Gating strategy for discrimination of 14 groups of phytoplankton species based on autofluorescence in a complex synthetic mix. (**a**) Ribbon plot demonstrating combined spectral signatures of 16 phytoplankton species across 7 excitation lasers. (**b**) Overlay plot of individual spectral signatures for each phytoplankton representative. (**c**) Gating strategy for discrimination of each subpopulation within the synthetic mix: 1—Chlorophyta (*Chlorella vulgaris* and *Acutodesmus obliquus*), 2—*Euglena sanguinea*, 3—*Peridinium* sp., 4—*Cryptomonas* sp., 5—*Fragilaria* sp., 6—*Haematococcus pluvialis*, 7—*Chroothece richteriana*, 8—*Porphyridium cruentum* cf., 9—filamentous Cyanobacteria (*Dolichospermum urugayensis* and *Nodularia sphaerocarpa*), 10—*Chroomonas* sp., 11—*Synechocystis* sp., 12—*Gloeobacter* violaceus, 13—*Microcystis* sp., and 14—*Synechococcus* sp.

To discriminate multiple components in a suspension, a mixture of unicellular cyanobacteria was used and recorded for further analysis (Figure 2). In addition, components of the mix represented variable taxonomic groups at the order level (*Microcystis* sp., belonging to Chroococcales), as well as members of the same order (*Synechococcus* sp., *Synechocystis* sp., belonging to Synechococcales). Individual spectral signatures were recorded prior to the mix; Figure 2b demonstrates an overlay plot of the acquired spectra across seven excitation lasers. As seen from the plot, *Microcystis* sp. (red) and *Synechococcus* sp. (blue) possess nearly identical spectral signatures, with slight differences in fluorescence intensity, while distinct region around channels 10–16 (566–646 nm) in the spectra for *Synechocystis* sp. (violet) was recorded. Based on the obtained information, a set of optimal VFs was chosen to first discriminate autofluorescent populations from unwanted events and to further separate these populations into respective picoplanktonic groups. The gating strategy is presented in Figure 2c, where a combination of [VF-647]_A vs. [VF-488]_A reveals two subpopulations, corresponding to *Synechocystis* sp. and population “A”. Due to the similarity in the spectra of *Microcystis* sp. and *Synechococcus* sp., subpopulation “A” was then differentiated based on SSC values. Identified populations could not be discriminated on FSC and SSC parameters only (Figure 2d). At this point, the maximum number of individual phytoplankton species that could be discriminated reached 14 (Figure 3).

### 3.3. Parallel Monitoring of Synthetic Phytoplankton Communities Using Full-Spectrum Cytometry and Long Sequencing

**Figure 4:**
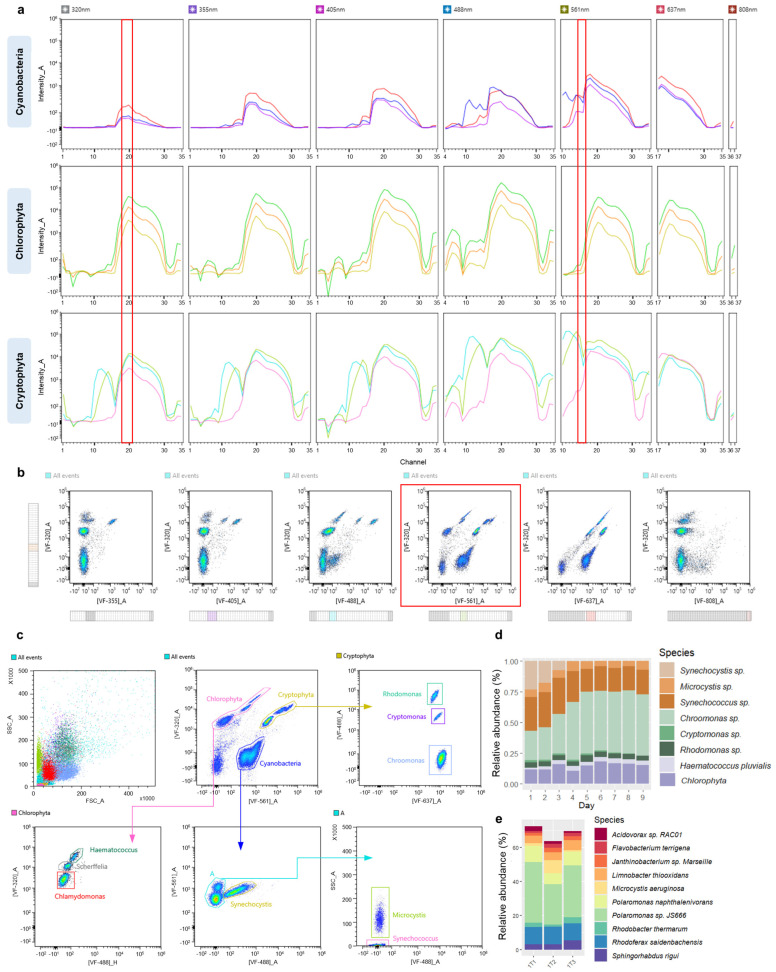
Tracking temporal changes in synthetic community composition. (**a**) Overlay plot of individual spectral signatures for each phytoplankton group representative (Cyanobacteria: violet—*Microcystis* sp., red—*Synechococcus* sp., blue—*Synechocystis* sp.; *Chlorophyta*: yellow—*Scherffelia dubia*, orange—*Chlamydomonas* sp., green—*Haematococcus pluvialis*; Cryptophyta: pink—*Chroomonas* sp., blue—*Rhodomonas* sp., green—*Cryptomonas* sp.); red regions indicate regions of interest, used for setting an optimal pair of VFs. (**b**) “Autofluorescence Finder” tool with VFs set to regions of interest, demonstrating the best separation of subpopulations on **[VF-320_A]** vs. **[VF-561_A]** plot (highlighted in red) (**c**) The gating strategy, composed of sequential sub-gating steps, used to identify phytoplankton subpopulations within the synthetic community; colored scatter dot plot demonstrates each of identified algal populations. (**d**) Changes in the relative abundance of the artificial mix subpopulations during the nine-day experiment. (**e**) Top ten most abundant bacterial species during the last day of the experiment in triplicates revealed through full-length 16S rRNA sequencing.

Next, full-spectrum cytometry’s applicability in monitoring the dynamics of synthetic phytoplankton consortium was assessed. For this purpose, an artificial mix experiment was set up; the synthetic mix included three representatives of major phytoplankton groups—Cyanobacteria, Chlorophyta, and Cryptophyta. Firstly, a library containing individual spectral signatures covering each phytoplankton species within the mix was constructed. Figure 4a presents nine spectral signatures across three phytoplankton groups: Cyanobacteria (*Microcystis* sp., *Synechococcus* sp., and *Synechocystis* sp.), Chlorophyta (*Chlamydomonas* sp., *Scherffelia dubia*, and *Haematococcus pluvialis*) and Cryptophyta (*Rhodomonas* sp., *Cryptomonas* sp., and *Chroomonas* sp.). As seen from the graph, regions unique for each of the groups are present, specifically between channels 10 and 15 (566–646 nm) of the spectra. This information was further utilized to define a set of optimal VF pairs to discriminate these populations in a complex mix. VFs were defined in a way that clusters subpopulations within the same group while maintaining the difference between the groups. A set of filters [VF-320]_A vs. [VF-561]_A (Figure 4b) was chosen as an optimal pair for the first step within the gating strategy (Figure 4c), where three groups are identified and discriminated from the rest of the unwanted events (background noise/cellular debris). Figure 4d summarizes averaged changes in the relative abundance of the components of the artificial mix throughout the experiment. Cryptophytes, specifically *Chroomonas* sp., were recorded as the dominant group within the mix starting from the fourth day, outnumbering cyanobacterial representatives. In addition to cytometric analysis, samples collected at the end of the experiment were also subjected to sequencing-based analysis. Figure 4e demonstrates microbial community composition across three replicates at species levels. The three most abundant phyla besides Cyanobacteria were Proteobacteria and Bacteroidetes, with *Polaromonas* sp. *JS666* (Betaproteobacteria) being the most abundant bacterial species. The second and third most abundant bacterial species identified were *Rhodoferax saidenbachensis* (Betaproteobacteria) and *Polaromonas naphthalenivorans* (Betaproteobacteria), respectively. The top ten most abundant bacterial species accounted for over 60% of total bacterial abundance within samples. A list of bacterial phyla and the top fifty species identified are listed in Tables S1 and S2. The results showed stability of bacterial composition in the artificial algal–microbial mix during a 9-day experiment, resulting in insignificant differences between sequenced triplicates.

**Figure 5:**
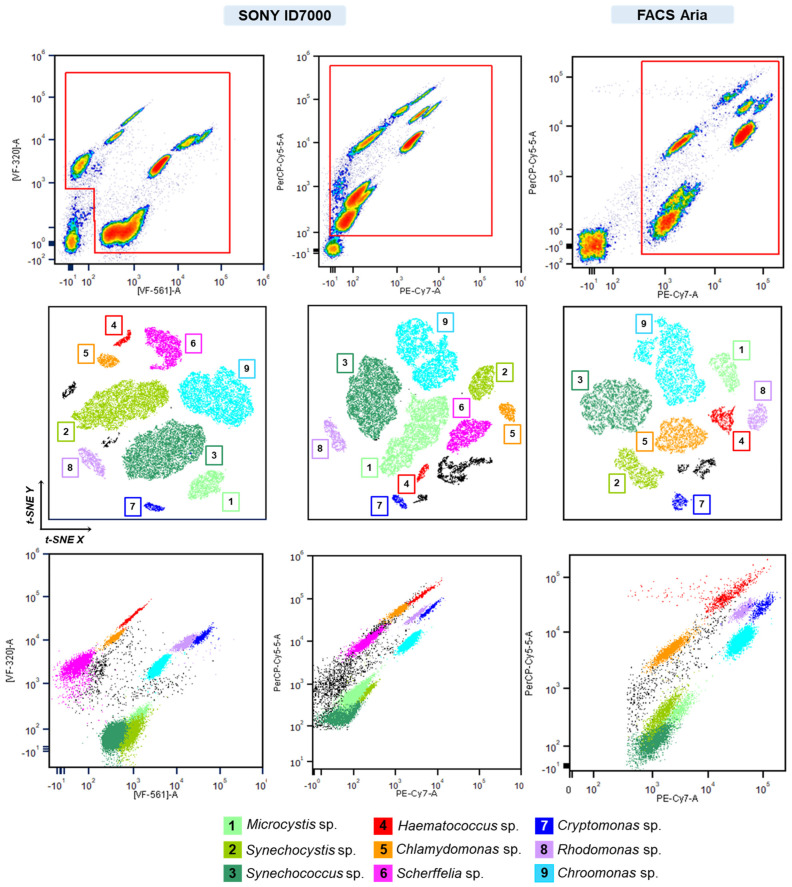
Identification of artificial mix subpopulations compared between SONY ID700 spectral cell analyzer and BD FACSAria SORP cytometer. Subpopulations of interest were first gated on the plots (red region) to eliminate debris. The first column represents data recorded on SONY ID7000 using a set of VFs best optimized for artificial mix discrimination. Opt-SNE demonstrates successful discrimination of the 9 subpopulations within the mix based on VF data. The second column represents data recorded on SONY ID7000 using a set of VFs matching optical filters used on BD FACSAria. Similarly, all 9 subpopulations were discriminated. The last column represents data recorded on BD FACSAria. Based on the optical configuration of the instrument, only 8 subpopulations were identified using opt-SNE.

**Figure 6:**
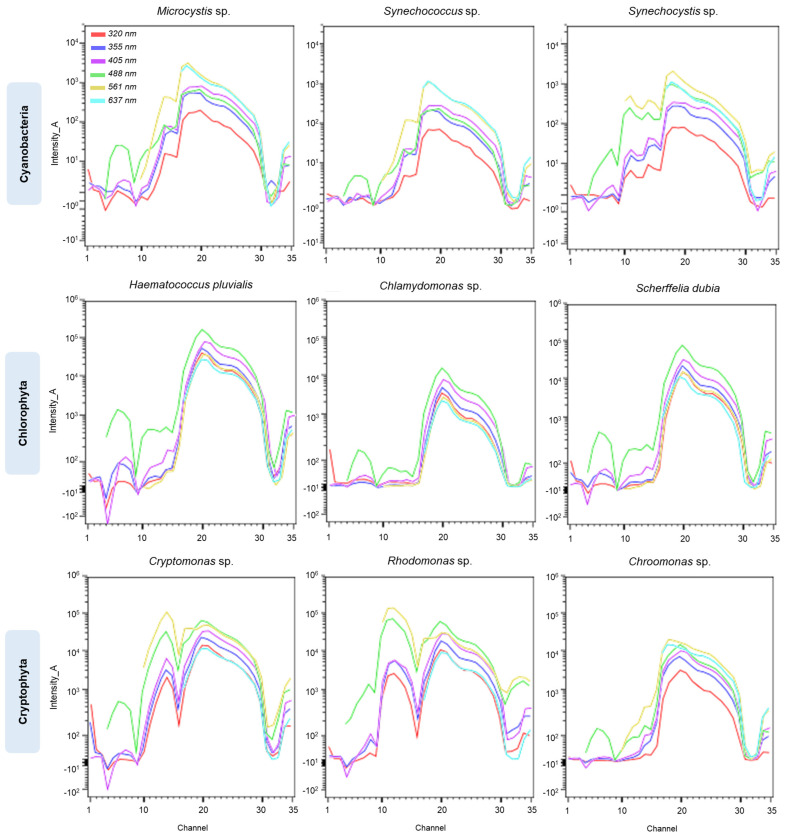
Overlay plots for each phytoplankton species within a synthetic mix across 7 excitation lasers to demonstrate the variability of emission signal for each excitation source. The first row demonstrates overlay plots for Cyanobacteria representatives, second—Chlorophyta, and third—Cryptophyta.

The next steps involved pairwise combinations of defined filters to discriminate the components of each of the subpopulations. As mentioned previously, due to the high similarity of spectra between *Microcystis* sp. and *Synechococcus* sp., side scatter (SSC) was used to differentiate these populations. A comparison of the data obtained on three instruments revealed limitations of some of the instruments (Figure 5). Opt-SNE dimensionality reduction algorithm was utilized to minimize “gating bias” and group data into clusters with similar characteristics in low-dimensional space. Three datasets were compared: synthetic mix data obtained from the ID7000 instrument using a set of pre-defined VFs and a set of VFs matching the filters used on FACSAria, and lastly, data obtained from FACSAria. Both sets of filters utilized on the ID7000 resulted in a clear separation of all nine subpopulations within the mix, while a single population of *Scherffelia dubia* was not possible to discriminate from the rest on the FACSAria instrument. These results demonstrate the higher flexibility a virtual filter system offers in the analysis of multi-component mixes. In addition to more precise population separation obtained with the ID7000 instrument, it was possible to track the abundance of nearly all mix components, as demonstrated in Figure S2. Overlay of temporal spectral signatures recorded for the period of 9 days for each mix member was used to assess the extent of signal intensity dispersion. As revealed through the coefficient of variation, regions most susceptible to differences in intensity generally included channels 1–13 (413.6–612.6 nm) and 30–34 (785.4–835.1 nm). High variation within these regions was also observed when comparing single spectral signatures between excitation lasers (Figure 6). 

## 4. Discussion

Phytoplankton are extraordinarily diverse, comprising various phylogenetic groups such as diatoms, dinoflagellates, haptophytes, and cyanobacteria. Developing spectral-based technologies for rapidly evaluating the dynamics of microalgal communities is crucial for ecological monitoring and engineering artificial microbial and algal communities. 

Flow cytometry, a well-established immunological and oncological research tool, has also been applied to phytoplankton analysis [37,38,39]. Early studies, which focused on the application of flow cytometry in aquatic research [40,41,42,43], have demonstrated cytometry to be a crucial tool in the study of bacterio- and phytoplankton. Early research demonstrated the analysis based on inherent properties, such as cell size and pigment fluorescence, including measurements of chlorophyll (Chl) and phycoerythrin (PE) emission [42]. Some of the advantages of this technique included but were not limited to long-term monitoring possibilities, high-throughput, and multiparameter analysis at the single-cell level. Recent studies have demonstrated the parallel application of conventional flow cytometry and sequencing in the analysis of pico- and phytoplanktonic communities in reservoir and coastal regions [44,45]. However, conventional flow cytometers, which rely on narrow band-pass filters to detect specific fluorophores, are limited by spectral overlap and the number of distinguishable fluorophores [46]. Alternative methods for the analysis of complex phytoplankton communities include microscopy and pigment-based chemotaxonomy, which are limited in terms of throughput and taxonomic resolution [47,48,49]. Algal community composition and parameters are traditionally defined by microscopy [50,51], which requires significant time; results vary between experts and do not allow for the determination of autotrophic picoplankton (<3 µm) [52]. A shortcoming of the pigment-based approach is that it does not provide taxonomic resolution beyond the class level [50].

Advancements in cytometry, particularly full-spectrum cytometry, present significant improvements in studying synthetic algal–microbial communities. This approach addresses limitations in traditional methods such as microscopy, pigment analysis, and conventional flow cytometry by enabling rapid, high-throughput, and label-free analysis of single algal cells from various taxonomic groups. By capturing the full emission spectra, spectral cytometry allows precise identification and enumeration of phytoplankton, facilitating a comprehensive understanding of community composition, structure, and dynamics. Additionally, it offers insights into responses to environmental stressors and enhances the characterization of microbiota associated with growing algae, advancing our understanding of phytoplankton ecology, synthetic biology, and evolution. One of the major challenges in conventional flow cytometry, specifically relevant to phytoplankton research, is the issue of autofluorescence. While in conventional flow cytometry, autofluorescence often interferes with fluorophore emission and is, therefore, an unwanted signal, spectral cytometry utilizes this phenomenon as a separate parameter(s) [53]. In addition, measuring the full spectrum of light emissions from each cell allows us to differentiate between various combinations of intrinsic fluorophores that conventional instruments cannot distinguish. This is particularly advantageous in phytoplankton research due to the high diversity of algal pigments and their overlapping spectral signatures. Alternately, approaches that provide high taxonomic resolution favor methods that allow for genus- to species-level characterization, such as amplicon sequencing [39]. 

In this work, we demonstrate the effectiveness of full-spectrum cytometry in differentiating between different algal taxa based on their autofluorescent signatures and light scattering parameters. Clear ability to detect autotrophic phytoplankton and separation between picocyanobacteria (*Synechococcus* sp. and *Synechocystis* sp.), as well as between closely related phytoplankton taxa (Synechococcales order), was demonstrated. Recorded spectral signatures were then utilized in the construction of a spectral library, which is a necessary step in monitoring synthetic communities over time. It was reported before that the spectral shape of the functional absorption spectra is remarkably constrained within major phylogenetic groups of phytoplankton, including diatoms, haptophytes, dinoflagellates, and chlorophytes [54], and variability is significantly smaller than the difference between groups. In our study, the emission spectra obtained from microalgae with the help of an ID7000 spectral cell analyzer highlight the ability to discriminate between multiple phytoplankton species in a synthetic mix, even in the presence of closely related taxa. This precision is achieved using optimized sets of virtual filters and gating strategies, which enable the clear separation of subpopulations. The temporal monitoring of synthetic phytoplankton communities further demonstrates the applicability of full-spectrum cytometry in tracking community dynamics and responses to environmental changes. Comparative analysis with other instruments, such as BD FACSAria SORP and ImageStreamX MarkII imaging flow cytometer, demonstrates the higher flexibility of the ID7000 spectral cell analyzer in terms of discrimination of subpopulations within a synthetic mix. 

The development of biological applications of full-spectrum cytometry in phytoplankton research is still in its early stages, with most published work focusing on clinical applications [27,31,55,56,57,58]. One of the limitations of this approach relates to the particle size range capabilities of spectral cytometry instrumentation similar to commercially available cytometers. As a result, bulky and lengthy plankton, such as many colonial and filamentous cyanobacteria, diatoms, etc., cannot be captured during the analysis. In addition, in situ measurement of planktonic cells is not available. In contrast, a range of other cytometric instruments, such as FlowCAM and CytoBuoy, designed for phytoplankton, have significantly extended particle size range, allowing analysis of particles of 500 µm width and over 1000 µm length [59,60,61]. Implementing full-spectrum cytometry in phytoplankton research requires reproducible protocols and standardization throughout the workflow, from sample collection and handling to data acquisition and analysis. Additionally, natural variability in phytoplankton populations, including cell size, shape, and pigment composition differences, requires optimized controls to minimize data variability. With the latest ID7000 software update, system standardization for data reproducibility became available. In phytoplankton research, recorded algal libraries containing unique spectral signatures can be utilized across different experiments, time points, and multiple instruments, minimizing data variability. Current work has resulted in a library that includes spectral signatures of over 35 unique phytoplankton species across different taxa. Moreover, important for the integrated species-level analysis of synthetic communities is early reported [36] species-level resolution of nanopore-based next-generation sequencing [62,63].

The stability of the microbial part of the artificial algal–microbial mix is in accordance with data from another group [64], which reported population stability with mutualistic co-culturing for up to 10 days. Microorganisms within the consortia could be unculturable; hence, culture-independent methods such as high-throughput sequencing and molecular fingerprinting are used to characterize the microbial part of consortia [65]. We suggest a nanopore-based sequencing at the species level to be performed not at the end of the experiment but in parallel with spectral cytometry evaluation or weekly.

## 5. Conclusions

In conclusion, by single-cell analysis and differentiating between different algal taxa based on their autofluorescent spectral signatures and light scattering parameters, full-spectrum cytometry overcomes the limitations of traditional methods such as microscopy, pigment analysis, and conventional cytometry. Its application extends to constructing spectral libraries for various phytoplankton groups and monitoring synthetic communities over time, providing detailed insights into community structure, dynamics, and responses to environmental stressors. The potential for parallel sequencing and associated microbial analysis at the multi-omics level is needed to elucidate the microbial functions occurring in artificial algal communities. This technology provides possibilities for a wide range of research applications, from addressing fundamental ecological and evolutionary questions to applied biology, thereby advancing environmental research.

## Figures and Tables

**Table 1:** List of algal cultures used for recordings during artificial mix experiment and library construction with ID7000 spectral cell analyzer.

Phylum	Order	Species	Habitat	Strain Number
Cyanobacteria	Nostocales	*Dolichospermum urugayensis*	Freshwater	2498
		*Nodularia sphaerocarpa*	Freshwater	50.79
	Gloeobacterales	*Gloeobacter violaceus*	Freshwater	35.87
	Chroococcales	*Microcystis* sp.	Freshwater	CCAC 7146B
	Synechococcales	*Synechococcus* sp.	Freshwater	CCAC 2944B
		*Synechocystis* sp.	Freshwater	CCAC 4059B
Chlorophyta	Chlorodendrales	*Scherffelia dubia*	Freshwater	CCAC 1398 B
	Chlamydomonadales	*Chlamydomonas* sp.	Freshwater	CCAC 4527 B
		*Haematococcus pluvialis*	Freshwater	CCAC 2072B
	Chlorellales	*Chlorella vulgaris*	Freshwater	211-12
	Sphaeropleales	*Acutodesmus obliquus*	Freshwater	22.81
Cryptophyta	Pyrenomonadales	*Chroomonas* sp.	Freshwater	CCAC 2614B
		*Rhodomonas* sp.	Freshwater	CCAC 1479B
	Cryptomonadales	*Cryptomonas* sp.	Freshwater	CCAC 2345B
Rhodophyta	Stylonematales	*Chroothece richteriana*	Marine	104.79
	Porphyridiales	*Porphyridium cruentum* cf.	Marine	CCAC 3771 B
Dinophyta	Peridiniales	*Peridinium* sp.	Freshwater	CCAC 1612 B
Euglenophyta	Euglenida	*Euglena sanguinea*	Freshwater	CCAC 3518 B
Bacillariophyta	Fragilariales	*Fragilaria* sp.	Freshwater	CCAC 5509 B

## Data Availability

Raw sequences obtained during the analysis were deposited in the National Center for Biotechnology Information (NCBI) Sequence Read Archive (SRA) under the BioProject ID PRJNA1131605. Spectral and cytometric data obtained during this study are available from the corresponding author upon reasonable request.

## Supplementary Materials

The following supporting information can be downloaded at https://www.mdpi.com/article/10.3390/cells13181552/s1: Table S1: Relative abundance (%) of bacterial phyla across triplicates identified using full-length 16S rRNA sequencing; Table S2: Relative abundance (%) of top fifty bacterial species across triplicates identified using full-length 16S rRNA sequencing; Figure S1: Gallery of images recorded during the artificial mix experiment with ImageStream MKII (Amnis-Cytek, Fremont, CA, USA) of phytoplankton species; Figure S2: Temporal overlay of spectral signatures recorded daily throughout the experiment.

## References

[1] Deng Y., Mauri M., Vallet M., Staudinger M., Allen R.J., Pohnert G. (2022). Dynamic Diatom-Bacteria Consortia in Synthetic Plankton Communities. Appl. Environ. Microbiol..

[2] Fu H., Uchimiya M., Gore J., Moran M.A. (2020). Ecological Drivers of Bacterial Community Assembly in Synthetic Phycospheres. Proc. Natl. Acad. Sci. USA.

[3] Hartwell L.H., Hopfield J.J., Leibler S., Murray A.W. (1999). From Molecular to Modular Cell Biology. Nature.

[4] Andersen R.A. (2005). Algal Culturing Techniques.

[5] De-Bashan L.E., Bashan Y., Moreno M., Lebsky V.K., Bustillos J.J. (2002). Increased Pigment and Lipid Content, Lipid Variety, and Cell and Population Size of the Microalgae Chlorella Spp. When Co-Immobilized in Alginate Beads with the Microalgae-Growth-Promoting Bacterium Azospirillum Brasilense. Can. J. Microbiol..

[6] Cho D.-H., Ramanan R., Heo J., Lee J., Kim B.-H., Oh H.-M., Kim H.-S. (2015). Enhancing Microalgal Biomass Productivity by Engineering a Microalgal–Bacterial Community. Bioresour. Technol..

[7] Xu Y., Wang Y., Yang Y., Zhou D. (2016). The Role of Starvation in Biomass Harvesting and Lipid Accumulation: Co-culture of Microalgae–Bacteria in Synthetic Wastewater. Environ. Prog. Sustain. Energy.

[8] Segev E., Wyche T.P., Kim K.H., Petersen J., Ellebrandt C., Vlamakis H., Barteneva N., Paulson J.N., Chai L., Clardy J. (2016). Dynamic Metabolic Exchange Governs a Marine Algal-Bacterial Interaction. eLife.

[9] Kolter R. (2024). Asking a Question. J. Bacteriol..

[10] Castle S.D., Grierson C.S., Gorochowski T.E. (2021). Towards an Engineering Theory of Evolution. Nat. Commun..

[11] Brenner K., You L., Arnold F.H. (2008). Engineering Microbial Consortia: A New Frontier in Synthetic Biology. Trends Biotechnol..

[12] Perera I.A., Abinandan S., Subashchandrabose S.R., Venkateswarlu K., Naidu R., Megharaj M. (2019). Advances in the Technologies for Studying Consortia of Bacteria and Cyanobacteria/Microalgae in Wastewaters. Crit. Rev. Biotechnol..

[13] Curry K.D., Wang Q., Nute M.G., Tyshaieva A., Reeves E., Soriano S., Wu Q., Graeber E., Finzer P., Mendling W. (2022). Emu: Species-Level Microbial Community Profiling of Full-Length 16S rRNA Oxford Nanopore Sequencing Data. Nat. Methods.

[14] Meirkhanova A., Zhumakhanova A., Len P., Schoenbach C., Levi E.E., Jeppesen E., Davidson T.A., Barteneva N.S. (2023). Dynamics of Associated Microbiomes during Algal Bloom Development: To See and to Be Seeing. bioRxiv.

[15] Poryvkina L., Babichenko S., Kaitala S., Kuosa H., Shalapjonok A. (1994). Spectral Fluorescence Signatures in the Characterization of Phytoplankton Community Composition. J. Plankton Res..

[16] Falkowski P.G., Oliver M.J. (2007). Mix and Match: How Climate Selects Phytoplankton. Nat. Rev. Microbiol..

[17] Roy S., Llewellyn C.A., Egeland E.S., Johnsen G. (2011). Phytoplankton Pigments: Characterization, Chemotaxonomy and Applications in Oceanography.

[18] Swan C.M., Vogt M., Gruber N., Laufkoetter C. (2016). A Global Seasonal Surface Ocean Climatology of Phytoplankton Types Based on CHEMTAX Analysis of HPLC Pigments. Deep Sea Res. Part I Oceanogr. Res. Pap..

[19] Liu J.-Y., Zeng L.-H., Ren Z.-H. (2021). The Application of Spectroscopy Technology in the Monitoring of Microalgae Cells Concentration. Appl. Spectrosc. Rev..

[20] Catlett D., Siegel D. (2018). Phytoplankton Pigment Communities Can Be Modeled Using Unique Relationships with Spectral Absorption Signatures in a Dynamic Coastal Environment. J. Geophys. Res. Oceans.

[21] Kramer S.J., Siegel D.A. (2019). How Can Phytoplankton Pigments Be Best Used to Characterize Surface Ocean Phytoplankton Groups for Ocean Color Remote Sensing Algorithms?. J. Geophys. Res. Oceans.

[22] Hochberg E.J., Atkinson M.J. (2003). Capabilities of Remote Sensors to Classify Coral, Algae, and Sand as Pure and Mixed Spectra. Remote Sens. Environ..

[23] Rodríguez Y.C., Gómez J.D., Sánchez-Carnero N., Rodríguez-Pérez D. (2017). A Comparison of Spectral Macroalgae Taxa Separability Methods Using an Extensive Spectral Library. Algal Res..

[24] Cawse-Nicholson K., Townsend P.A., Schimel D., Assiri A.M., Blake P.L., Buongiorno M.F., Campbell P., Carmon N., Casey K.A., Correa-Pabón R.E. (2021). NASA’s Surface Biology and Geology Designated Observable: A Perspective on Surface Imaging Algorithms. Remote Sens. Environ..

[25] Futamura K., Sekino M., Hata A., Ikebuchi R., Nakanishi Y., Egawa G., Kabashima K., Watanabe T., Furuki M., Tomura M. (2015). Novel Full-spectral Flow Cytometry with Multiple Spectrally-adjacent Fluorescent Proteins and Fluorochromes and Visualization of in Vivo Cellular Movement. Cytom. Part A.

[26] Robinson J.P. (2022). Flow Cytometry: Past and Future. Biotechniques.

[27] Dott T., Culina S., Chemali R., Mansour C.A., Dubois F., Jagla B., Doisne J.M., Rogge L., Huetz F., Jönsson F. (2024). Standardized High-dimensional Spectral Cytometry Protocol and Panels for Whole Blood Immune Phenotyping in Clinical and Translational Studies. Cytom. Part A.

[28] Barteneva N.S., Dashkova V., Vorobjev I. (2019). Probing Complexity of Microalgae Mixtures with Novel Spectral Flow Cytometry Approach and “Virtual Filtering”. bioRxiv.

[29] Barteneva N.S., Kussanova A., Dashkova V., Meirkhanova A., Vorobjev I.A. (2023). Using Virtual Filtering Approach to Discriminate Microalgae by Spectral Flow Cytometer. Spectral and Imaging Cytometry: Methods and Protocols.

[30] Jeppesen E., Søndergaard M., Jensen J.P., Havens K.E., Anneville O., Carvalho L., Coveney M.F., Deneke R., Dokulil M.T., Foy B. (2005). Lake Responses to Reduced Nutrient Loading—An Analysis of Contemporary Long-term Data from 35 Case Studies. Freshwater Biol..

[31] Wanner N., Barnhart J., Apostolakis N., Zlojutro V., Asosingh K. (2022). Using the Autofluorescence Finder on the Sony ID7000TM Spectral Cell Analyzer to Identify and Unmix Multiple Highly Autofluorescent Murine Lung Populations. Front. Bioeng. Biotechnol..

[32] Geng H., Sale K.L., Tran-Gyamfi M.B., Lane T.W., Yu E.T. (2016). Longitudinal Analysis of Microbiota in Microalga Nannochloropsis Salina Cultures. Microb. Ecol..

[33] Barteneva N.S., Vorobjev I.A. (2023). Spectral and Imaging Cytometry: Methods and Protocols.

[34] Frank J.A., Reich C.I., Sharma S., Weisbaum J.S., Wilson B.A., Olsen G.J. (2008). Critical Evaluation of Two Primers Commonly Used for Amplification of Bacterial 16S rRNA Genes. Appl. Environ. Microbiol..

[35] Mao D.P., Zhou Q., Chen C.Y., Quan Z.X. (2012). Coverage evaluation of Universal Bacterial Primers Using the metagenomic datasets. BMC Microbiol..

[36] Len P., Meirkhanova A., Nugumanova G., Cestaro A., Jeppesen E., Vorobjev I.A., Donati C., Barteneva N.S. (2023). Species-Level Classification Provides New Insights into the Biogeographical Patterns of Microbial Communities in Shallow Saline Lakes. bioRxiv.

[37] Trask B.J., Van den Engh G.J., Elgershuizen J.H.B.W. (1982). Analysis of Phytoplankton by Flow Cytometry. Cytometry.

[38] Yentsch C.M., Horan P.K., Muirhead K., Dortch Q., Haugen E., Legendre L., Murphy L.S., Perry M.J., Phinney D.A., Pomponi S.A. (1983). Flow Cytometry and Cell Sorting: A Technique for Analysis and Sorting of Aquatic Particles. Limnol. Oceanogr..

[39] Sosik H.M., Olson R.J., Armbrust E.V. (2010). Flow Cytometry in Phytoplankton Research. Chlorophyll a Fluorescence in Aquatic Sciences: Methods and Applications.

[40] Paau A.S., Oro J., Cowles J.R. (1978). Application of Flow Microflorometry to the Study of Algal Cells and Isolated Chloroplasts. J. Exp. Bot..

[41] Hutter K.J., Eipel H.E. (1978). Flow Cytometric Determinations of Cellular Substances in Algae, Bacteria, Moulds and Yeasts. Antonie Leeuwenhoek.

[42] Phinney D.A., Cucci T.L. (1989). Flow Cytometry and Phytoplankton. Cytometry.

[43] Li W.K.W., Dickie P.M. (2001). Monitoring Phytoplankton, Bacterioplankton, and Virioplankton in a Coastal Inlet (Bedford Basin) by Flow Cytometry. Cytometry.

[44] Ning M., Li H., Xu Z., Chen L., He Y. (2021). Picophytoplankton Identification by Flow Cytometry and High-Throughput Sequencing in a Clean Reservoir. Ecotoxicol. Environ. Saf..

[45] Robicheau B.M., Tolman J., Bertrand E.M., LaRoche J. (2022). Highly-resolved Interannual Phytoplankton Community Dynamics of the Coastal Northwest Atlantic. ISME Commun..

[46] Vorobjev I.A., Kussanova A., Barteneva N.S. (2023). Development of Spectral Imaging Cytometry. Spectral and Imaging Cytometry: Methods and Protocols.

[47] Culverhouse P.F. (2007). Human and Machine Factors in Algae Monitoring Performance. Ecol. Inform..

[48] Willén E. (2000). Phytoplankton in Water Quality Assessment–an Indicator Concept. Hydrological and Limnological Aspects of Lake Monitring.

[49] Catherine A., Escoffier N., Belhocine A., Nasri A.B., Hamlaoui S., Yéprémian C., Bernard C., Troussellier M. (2012). On the Use of the FluoroProbe^®^, a Phytoplankton Quantification Method Based on Fluorescence Excitation Spectra for Large-Scale Surveys of Lakes and Reservoirs. Water Res..

[50] Tamm M., Freiberg R., Tõnno I., Nõges P., Nõges T. (2015). Pigment-based Chemotaxonomy-a Quick Alternative to Determine Algal Assemblages in Large Shallow Eutrophic Lake?. PLoS ONE.

[51] Simmons L.J., Sandgren C.D., Berges J.A. (2016). Problems and Pitfalls in Using HPLC Pigment Analysis to Distinguish Lake Michigan Phytoplankton Taxa. J. Great Lakes Res..

[52] Havskum H., Schlüter L., Scharek R., Berdalet E., Jacquet S. (2004). Routine quantification of phytoplankton groups microscopy or pigment analyses?. Mar. Ecol. Prog. Ser..

[53] Jameson V.J., Luke T., Yan Y., Hind A., Evrard M., Man K., Mackay L.K., Kallies A., Villadangos J.A., McWilliam H.E.G. (2022). Unlocking Autofluorescence in the Era of Full Spectrum Analysis: Implications for Immunophenotype Discovery Projects. Cytom. Part A.

[54] Sommeria-Klein G., Watteaux R., Ibarbalz F.M., Pierella Karlusich J.J., Iudicone D., Bowler C., Morlon H. (2021). Global Drivers of Eukaryotic Plankton Biogeography in the Sunlit Ocean. Science.

[55] Camp B., Jorde I., Sittel F., Pausder A., Jeron A., Bruder D., Schreiber J., Stegemann-Koniszewski S. (2024). Comprehensive Analysis of Lung Macrophages and Dendritic Cells in Two Murine Models of Allergic Airway Inflammation Reveals Model- and Subset-Specific Accumulation and Phenotypic Alterations. Front. Immunol..

[56] Bourdely P., Petti L., Khou S., Meghraoui-Kheddar A., Elaldi R., Cazareth J., Mossadegh-Keller N., Boyer J., Sieweke M.H., Poissonnet G. (2022). Autofluorescence Identifies Highly Phagocytic Tissue-resident Macrophages in Mouse and Human Skin and Cutaneous Squamous Cell Carcinoma. Front. Immunol..

[57] Schmutz S., Commere P.H., Montcuquet N., Cumano A., Ait-Mansour C., Novault S., Hasan M. (2024). Beyond 40 fluorescent probes for deep phenotyping of blood mononuclear cells, using spectral technology. Front. Immunol..

[58] Peixoto M.M., Soares-da-Silva F., Schmutz S., Mailhe M., Novault S., Cumano A., Ait-Mansour C. (2022). Identification of Fetal Liver Stroma in Spectral Cytometry Using the Parameter Autofluorescence. Cytom. Part A.

[59] Dubelaar G.B., Groenewegen A.C., Stokdijk W., Van Den Engh G., Visser J.W. (1989). Optical Plankton Analyser: A Flow Cytometer for Plankton Analysis, II: Specifications. Cytometry.

[60] Sieracki C.K., Sieracki M.E., Yentsch C.S. (1998). An Imaging-in-Flow System for Automated Analysis of Marine Microplankton. Mar. Ecol. Prog. Ser..

[61] Dubelaar G., Gerritzen P. (2000). CytoBuoy: A Step Forward towards Using Flow Cytometry in Operational Oceanography. Sci. Mar..

[62] Matsuo Y., Komiya S., Yasumizu Y., Yasuoka Y., Mizushima K., Takagi T., Kryukov K., Fukuda A., Morimoto Y., Naito Y. (2021). Full-length 16S rRNA Gene Amplicon Analysis of Human Gut Microbiota using MinION™ Nanopore Sequencing Confers Species-level Resolution. BMC Microbiol..

[63] Szoboszlay M., Schramm L., Pinzauti D., Scerri J., Sandionigi A., Biazzo M. (2023). Nanopore is Preferable over Illumina for 16S Amplicon Sequencing of the Gut Microbiota When Species-level Taxonomic Classification, Accurate Estimation of Richness, or Focus on Rare Taxa is Required. Microorganisms.

[64] Li X., Zhou Z., Li W., Yan Y., Shen X., Wang J., Sun X., Yuan Q. (2022). Design of Stable and Self-Regulated Microbial Consortia for Chemical Synthesis. Nat. Commun..

[65] De Roy K., Marzorati M., Van den Abbeele P., Van de Wiele T., Boon N. (2014). Synthetic Microbial Ecosystems: An Exciting Tool to Understand and Apply Microbial Communities. Environ. Microbiol..

