# Supplementary material for: Spectral Algal Fingerprinting and Long Sequencing in Synthetic Algal–Microbial Communities
Source: Cells. 2024 Sep 14;13(18):1552. doi: 10.3390/cells13181552 (PMC11430485; doi:10.3390/cells13181552)
Supplement: Supplementary file 1 [file cells-13-01552-s001.zip › cells-3156882-supplementary.pdf]

Spectral Algal Fingerprinting and Long Sequencing in Synthetic Algal–Microbial Communities

Ayagoz Meirkhanova [1], Sabina Marks [2], Nicole Feja [2], Ivan A. Vorobjev [1,3], Natasha S. Barteneva [1,4,*]

1 School of Science and Humanities, Nazarbayev University, Astana 010000, Kazakhstan; ayagoz.meirkhanova@nu.edu.kz (A.M.); ivan.vorobyev@nu.edu.kz (I.A.V.)
2 Faculty of Biology, University of Duisburg-Essen, Campus Essen, 45141 Essen, Germany; sabina.marks@uni-due.de (S.M.); nicole.feja@uni-due.de (N.F.)
3 National Laboratory Astana, Nazarbayev University, Astana 010000, Kazakhstan
4 The Environmental Research and Efficiency Cluster, Nazarbayev University, Astana 010000, Kazakhstan
* Correspondence: natalie.barteneva@nu.edu.kz

Supplementary material

Table S1. Relative abundance (%) of bacterial phyla across triplicates identified using full-length 16S rRNA sequencing

Phylum	1T1	1T2	1T3
Verrucomicrobia	0,00	0,04	0,01
Cyanobacteria	2,60	18,98	2,86
Gemmatimonadetes	0,33	0,06	0,46
Actinobacteria	0,00	0,00	0,01
Proteobacteria	87,39	66,46	86,62
Firmicutes	0,11	0,11	0,13
Bacteroidetes	9,57	14,35	9,91

Table S2. Relative abundance (%) of top fifty bacterial species across triplicates identified using full-length 16S rRNA sequencing

Species	1T1	1T2	1T3
Stanieria cyanosphaera	0,06	0,54	0,09
Methylotenera versatilis	0,34	0,28	0,30
Sphingorhabdus planktonica	0,67	0,32	1,01
Microcystis aeruginosa	1,55	7,54	0,90
Porphyrobacter colymbi	0,26	0,13	0,67
Geitlerinema sp. PCC 7407	0,08	0,91	0,14
Undibacterium terreum	0,14	0,64	0,23
Pedobacter pituitosus	0,44	0,24	0,36
Sphingorhabdus rigui	3,13	3,19	5,52
Limnobacter thiooxidans	4,51	4,75	6,00
Undibacterium seohonense	0,76	0,92	0,81
Rhodoferax saidenbachensis	10,22	10,19	9,98

Sporocytophaga myxococcoides	0,24	0,36	0,26
Sphingobacterium sp. ML3W	0,38	1,20	0,43
Blastomonas fulva	0,21	0,18	0,50
Limnohabitans sp. 63ED37-2	1,37	0,45	1,58
Sediminibacterium aquarii	0,19	0,34	0,39
Gemmatimonas aurantiaca	0,31	0,05	0,42
Stanieria sp. NIES-3757	0,07	1,40	0,20
Acidovorax sp. RAC01	2,76	1,58	1,11
Pedobacter petrophilus	0,20	0,29	0,18
Sandarakinorhabdus cyanobacteriorum	0,45	1,03	0,48
Pseudomonas poae	0,72	0,44	1,00
Derxia gummosa	0,71	0,39	0,79
Massilia violaceinigra	0,38	0,78	0,71
Pedobacter mongoliensis	0,53	0,81	0,51
Sandarakinorhabdus limnophila	0,52	0,34	0,60
Polaromonas naphthalenivorans	9,54	6,50	8,03
Pseudomonas sp. LG1E9	0,65	0,22	0,34
Polaromonas sp. SP1	1,38	1,56	1,30
Mucilaginibacter sp. HYN0043	0,15	0,23	0,15
Pedobacter changchengzhani	0,28	0,59	0,25
Rhodobacter thermarum	2,39	1,39	3,41
Collimonas arenae	0,40	0,56	0,56
Polaromonas sp. JS666	35,64	23,65	30,48
Adhaeribacter aquaticus	0,69	0,33	0,51
Sphingobacterium faecium	0,24	0,37	0,20
Janthinobacterium sp. Marseille	1,54	2,62	2,33
Flavobacterium terrigena	1,14	2,33	1,94
Polaromonas jejuensis	0,61	0,09	0,43
Azospirillum thiophilum	2,21	0,45	1,48
Solitalea koreensis	1,53	1,30	1,31
beta proteobacterium CB	1,15	0,54	1,58
Azospirillum sp. TSH100	0,61	0,10	0,42
Pseudobacter ginsenosidimutans	0,76	1,59	0,67
Candidatus Atelocyanobacterium thalassa	0,16	2,33	0,33
cyanobacterium endosymbiont of Epithemia turgida	0,16	2,06	0,37
Herbaspirillum seropedicae	0,31	0,36	0,45
Loriellopsis cavernicola	0,16	1,87	0,32
Solitalea canadensis	0,94	1,64	0,87

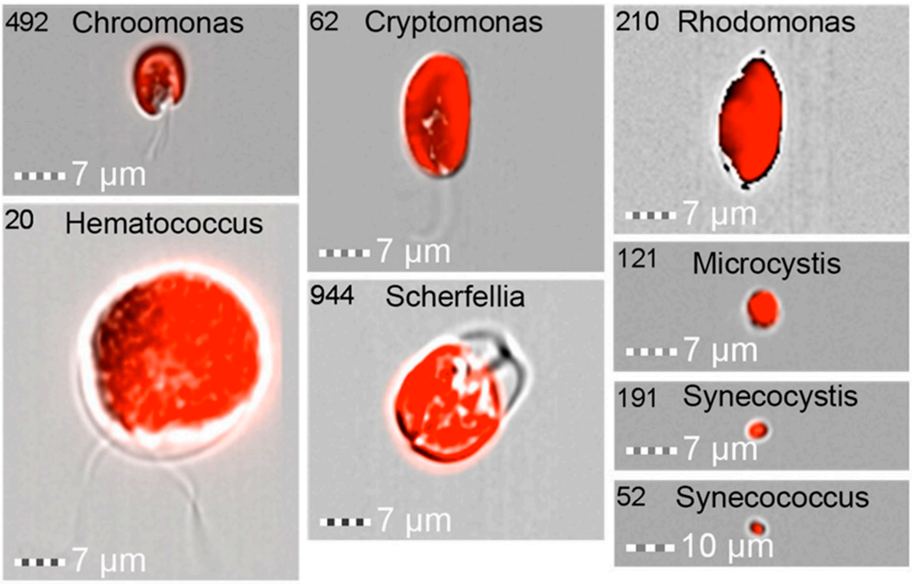

Figure S1. Gallery of images recorded during the artificial mix experiment. with ImageStream MKII (Amnis-Cytek, Fremont, CA, USA) of phytoplankton species.

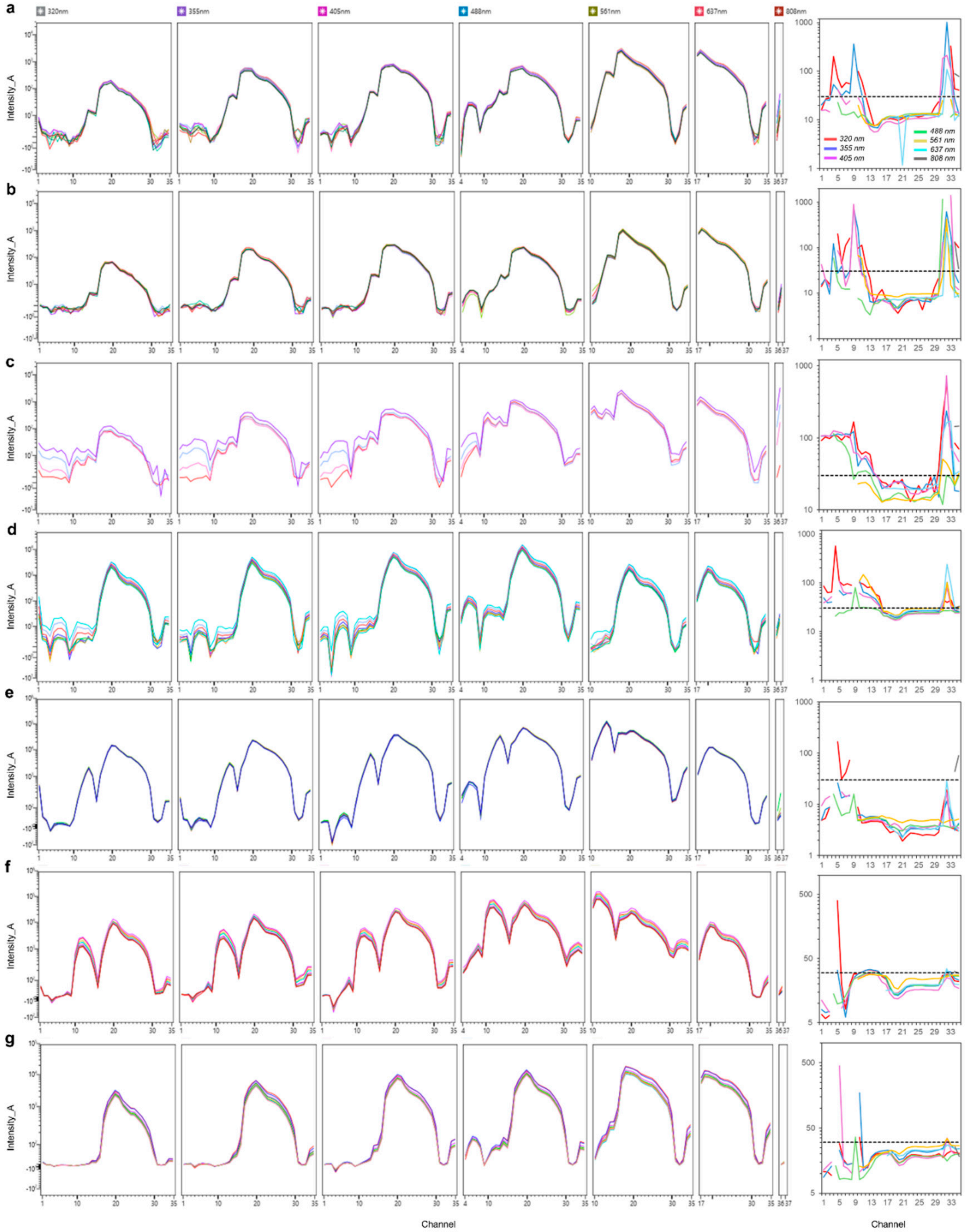

Figure S2. Temporal overlay of spectral signatures recorded daily throughout the experiment. Individual spectral signatures for each member of the synthetic mix ((a) – *Microcystis* sp., (b) - *Synechococcus* sp., (c) – *Synechocystis* sp., (d) – *Chlamydomonas* sp., (e) - *Cryptomonas* sp., (f) – *Rhodomonas* sp., (g) – *Chroomonas* sp.) recorded over the period of 9 days overlayed to demonstrate temporal changes in emission intensity. To display signal dispersion, coefficient of variation was calculated and plotted across all detection channels for each of the lasers (lasers are represented by different colors: red – 320nm, blue – 355nm, violet – 405nm, green – 488nm, yellow – 561nm, light-blue – 637nm, grey – 808nm). A threshold line at 30% was plotted for additional reference.
